# Boosting Power Conversion Efficiency of Quantum Dot-Sensitized Solar Cells by Integrating Concentrating Photovoltaic Concept with Double Photoanodes

**DOI:** 10.1186/s11671-020-03424-8

**Published:** 2020-09-29

**Authors:** Pei Xu, Xiaopeng Chang, Runru Liu, Liying Wang, Xuesong Li, Xueyu Zhang, Xijia Yang, Dejun Wang, Wei Lü

**Affiliations:** 1 Key Laboratory of Materials Design and Quantum Simulation, College of Science, Changchun University, Changchun, 130012 People’s Republic of China; 2 Key Laboratory of Advanced Structural Materials, Ministry of Education & Advanced Institute of Materials Science, Changchun University of Technology, Changchun, 130012 People’s Republic of China

**Keywords:** Quantum dot-sensitized solar cells, Concentrating photovoltaic, Double photoanodes, Mesh counter electrode

## Abstract

Despite great efforts dedicated to enhance power conversion efficiency (PCE) of quantum dot-sensitized solar cells (QDSSCs) in the past two decades, the efficiency of QDSSCs is still far behind its theoretical value. The present approaches for improving PCE are mainly focused on tailoring the bandgap of QDs to broadening light-harvesting and optimizing interfaces of component parts. Herein, a new solar cell architecture is proposed by integrating concentrating solar cell (CPV) concept into QDSSCs with double photoanode design. The Cu_2_S mesh is used as a counter electrode and sandwiched between two photoanodes. This designed battery structure can increase the PCE by 260% compared with a single photoanode. With the most extensively used CdS/CdSe QD sensitizers, a champion PCE of 8.28% (*V*_oc_ = 0.629 V, *J*_sc_ = 32.247 mA cm^−^2) was achieved. This is mainly due to the increase in *J*_sc_ due to the double photoanode design and adoption of the CPV concept. In addition, another reason is that concentrated sunshine illumination induced a photothermal effect, accelerating the preceding chemical reactions associated with the conversion of polysulfide species. The cell fabrication and design reported here provides a new insight for further development of QDSSCs.

## Introduction

As a kind of promising and comparably economic photoelectrical conversion device, quantum dot-sensitized solar cells (QDSSCs) have attracted great attention due to their high theoretical power conversion efficiency (PCE) [1, 2]. QDSSCs inherit the structure of dye-sensitized solar cells, including photoanode (typically, a layer of porous oxide semiconductor with a wide bandgap covered by semiconductor QDs as sensitizers), liquid electrolyte, and counter electrode. In order to enhance cell PCE for potential commercial application, numerous strategies have been investigated mainly from material viewpoints, involving synthesizing new QD sensitizers for broadening light-harvesting range; preparing photoanodes with different nanostructures such as porous film [3], nanotube [4, 5], and nanorod for better electron extracting [6, 7]; fabricating noble metal-doped photoanode using localized surface plasmon resonance [8, 9]; developing sulfide and other counters to replace Pt counter; synthesizing different electrolytes; and so on.

One of the most important factors limiting the photoelectric conversion efficiency of QDSSCs is the narrow absorbing range of solar radiation [10]. The absorption region is strongly dependent upon photoanode thin film and QDs. Narrowing bandgap of QDs is an effective way for broadening light-harvesting range. Zhong et al. synthesized Zn–Cu–In–Sn alloyed QDs and greatly improved the PCE of QDSSCs, setting several PCE records of QDSSCs [11–13]. Currently, the reported highest PCE of QDSSCs is 14.02% achieved by Zhao et al. [14]. In their work, Zn–Cu–In–Sn alloyed QDs were used as a sensitizer, and the counter electrode (CE) is CuS doped with carbon nanotubes and graphene, which initiated a new level of the PCE of QDSSCs. However, excessively decreasing the bandgap to enhance sunlight harvesting will induce the open-circuit voltage loss and decrease the device performance due to the downshift of the conduction band edge.

To further improve cell performance, device configuration has been taken into account. In dye-sensitized solar cells and colloidal quantum dot solar cells [15], tandem structures have already been used as a significant approach to break through the PCE limitations [16, 17]. For most tandem structures of dye-sensitized solar cells, a semi-transparent Pt on conductive glass or Pt mesh is applied in the middle of the cell as the counter electrode [18]. TiO_2_ films are separated into two or more layers sensitized by the same or different dyes, which can broaden the spectral response and thereby significantly improve the cell performance [19–21]. For QDSSCs, Meng et al. design a double photoanode structure with a semi-transparent mesh-structured Cu_2_S counter electrode sandwiched between two TiO_2_ photoelectrodes. Under the one sun illumination from the top electrode, the optimized cell shows a 12% increase in PCE [22]. In their work, the tandem structure was used in QDSSCs for the first time. However, the light utilization efficiency of the battery tandem structure is limited. Xu et al. demonstrated the NIR light-enhanced polysulfide reduction at the electrode–electrolyte interface by illuminating the CuS counter electrode with NIR light, showing a 15% increase in PCE, which is attributed to photothermal effect and plasmonic resonance absorption [23].

Thanks to the great efforts in the past several decades, the PCE of QDSSCs is allowed to be comparative with that of the parallel device, dye-sensitized solar cell. However, the efficiency of QDSSCs is still far behind its theoretical value, and it still remains to be solved for commercial application. Therefore, exploring new strategy to boost PCE of QDSSCs is still an urgent task. Herein, we integrate concentrating photovoltaic concept into QDSSCs with double porous TiO_2_ photoanodes and Cu_2_S mesh counter electrode design [24], achieving a 260% increase in PCE compared with the traditional single photoanode device. With the most extensively used CdS/CdSe QD sensitizers, a champion PCE of 8.28% (*V*_oc_ = 0.629 V, *J*_sc_ = 32.247 mA cm^−2^) is achieved.

**Fig. 1:**
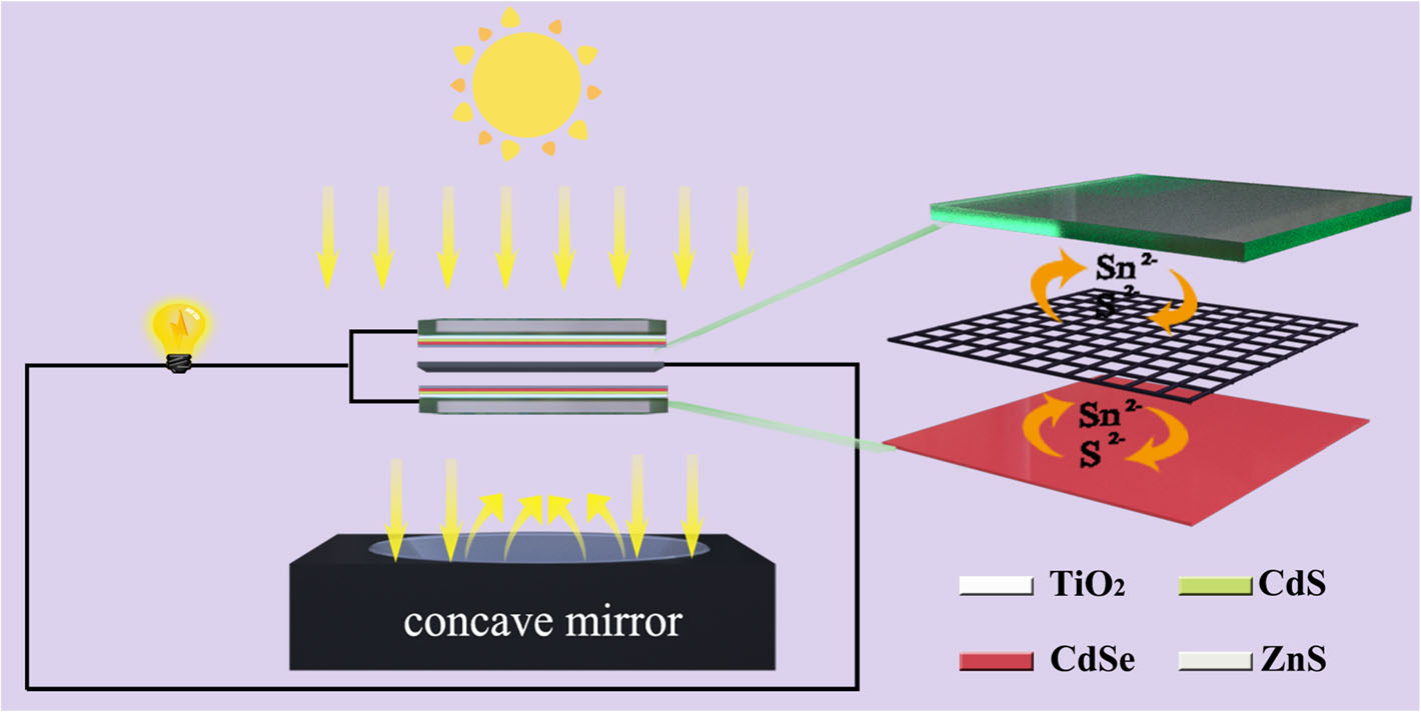
Schematic diagram of the cell design in the present work

Light management as an important technology to improve the conversion efficiency in solar cells aims to increase the photon flux received by solar cells [25]. The light-trapping structure is a commonly used method for light management, including mirrors [26, 27], Lambertian surfaces [28], and textured surfaces [29, 30]. By the light-trapping effect, the optical path length is increased, thereby improving the PCE of solar cells. One of the basic light-trapping structures is to prepare a mirror on the back surface of the solar cell, and the reflectivity of the mirror can be as high as 95% [31]. Inspired by the above discussion, Fig. 1 illustrates the device architecture used in the present work. Double TiO_2_ photoanode design is adopted, which is separated by Cu_2_S mesh as the counter electrode. The thickness of the TiO_2_ photoanode is about 4.5 μm, which can be seen in Fig. S1. The top cell and the bottom cell use the same material and structure, so it is not necessary to consider the lattice constant and expansion coefficient of different semiconductor materials in the two photoanodes. The Cu_2_S grown on Cu mesh as the counter electrode allows the transmitted light from the top cell to arrive bottom photoanode. The wide bandgap materials at the top filter out high energy photons, and the low energy photons pass through them and then absorbed by the narrow bandgap materials at the bottom cell. Apparently, the PCE of the double photoanode structure is higher than that of the single photoanode structure because the double photoanode structure can capture more light and thus increase current density. However, illuminating only from the top direction induces just an 18% increase in PCE compared with a single photoanode device as shown later, which is due to the strong absorption and reflection of sunshine by the top cell, leaving inadequate light density for the bottom cell [22].

Considering that the captured light of the bottom cell is limited, we introduce the concept of concentrating photovoltaic cell (CPV) into the current device [32, 33] and set a parabolic reflector under the bottom photoanode. CPV systems use optical elements to focus light on a small area of solar cells, which improves the efficiency of photovoltaic technology while minimizing costs. The reduction of cell area can make the cost of CPV compete with flat panel photovoltaic. As shown in Fig. 1, the parabolic reflector makes focused light on the bottom photoanode. By controlling the distance between the reflector and the bottom photoanode, the active area could be adjusted to match the area of the cell. In addition, the augmented light could transmit and get to the top photoanode, providing additional excitation energy for light harvesting. Of course, the photothermal effect caused by CPV should have an impact on the PV performance [34], which will be discussed in detail later.

## Methods

### Materials

Titanium dioxide (99.8%, 10–25 nm, TiO_2_) and sodium sulfite (AR, Na_2_SO_3_) were supplied from Aladdin. Fluorine-doped tin oxide conducting glass (FTO, thickness 1.6 mm, transmittance 83%, sheet resistance 15/square) was purchased from Zhuhai Kaivo Optoelectronic Technology Co., Ltd. (China). Methanol (AR, CH_3_OH), ethanol (AR, CH_3_CH_2_OH), and acetone (AR, CH_3_OCH_3_) were supplied by Beijing Chemical Works (China). Ethylenediamine (AR, C_2_H_8_N_4_) and selenium (AR, Se) were purchased from Tianjin Guangfu Fine Chemical Research Institute (China). Hydrochloric acid (36%, HCl), ethyl cellulose ethoce (AR, [C_6_H_7_O_2_(OC_2_H_5_)_3_]_*n*_), alpha-terpineol (AR, C_10_H_8_O), cadmium acetate (AR, Cd(CH_3_COOH)_2_), zinc acetate (AR, Zn(CH_3_COOH)_2_), sodium sulfide (AR, Na_2_S∙9H_2_O), sulfur (AR, S), and thiourea (AR, CH_4_N_2_S) were supplied by Sinopharm Chemical Reagent Co., Ltd. (China). Nitrilotriacetic acid trisodium (98%, C_6_H_9_NNa_3_O_6_) was purchased from TCI (Shanghai) Chemical Industry Development Co., Ltd. (China). Brass mesh was supplied by Hebei Xingheng Wire Mesh Products Co., Ltd. (China).

### Preparation of the Cu_2_S Counter Electrode

The brass mesh was soaked in HCl (36%) for 2 h at 70 °C to remove the zinc on the surface of the Cu mesh. The copper mesh was rinsed with deionized water and dried at room temperature after removing the copper mesh from the solution. Then, the Cu mesh was immersed into an aqueous solution containing 1 M Na_2_S and 1 M S for 5 s to get flake Cu_2_S, followed by washing with deionized water and drying at room temperature. To prepare granular Cu_2_S, the Cu mesh treated with HCl (36%) was immersed in a mixed solution of CH_4_N_2_S (0.01 M) and C_2_H_8_N_4_ (0.4 ml) for 24 h, followed by washing with deionized water and drying at room temperature.

### Preparation of CdS/CdSe QDs Co-sensitized TiO_2_ Photoanode

TiO_2_ mesoporous film was prepared by spin-coating a paste containing TiO_2_ (0.01 M), EC (0.4 g), C_10_H_8_O (3.245 g), and CH_3_CH_2_OH (8.5 ml) on cleaned FTO substrates, which was dried in an oven at 60 °C and followed by annealing at 450 °C for 30 min to remove the organic solvents. The rotation speed of the spin coater is 7500 rpm for 30 s. The successive ionic layer adsorption and reaction (SILAR) process was employed to deposit the CdS QDs. Typically, the TiO_2_ film was alternately dipped into methanol solution of Cd(CH_3_COOH)_2_ (0.12 M) and Na_2_S solution (0.02 M with methanol and deionized water 1:1 v/v) for 30 S in each cycle for total 5 cycles. CdSe QDs were coated by chemical bath deposition (CBD) method. In detail, a solution containing Cd^2+^ and Se^2−^ source was prepared by the following method. 1.55 g Na_2_SO_3_ was dissolved in 25 ml deionized water. 0.155 g Se powder as Se^2−^ source was added into the above solution. The obtained solution was heated with an oil bath at 125 °C for 3 h under stirring condition. The Cd^2+^ solution was prepared by mixing 25 ml nitrilotriacetic acid deionized water solution (120 mM) and 25 ml Cd(CH_3_COO)_2_ deionized water solution (80 mM). Then, the prepared Se^2−^ solution and Cd^2+^ solution were mixed together. The as-prepared CdS-coated photoanodes were put into the above mixed solution at 24 °C for 2 h in the dark. For the ZnS passivation, the sensitized films were deposited by dipping into 0.1 M Zn(CH_3_COO)_2_ and 0.1 M Na_2_S (with methanol and water 1:1 v/v) solutions for 1 min alternately and 4 cycles. The active area of the solar cell is 0.25 cm^2^. The polysulfide electrolyte (2 M Na_2_S and 2 M S solution with methanol and deionized water 7:3 v/v) was transfused into the cell via injector.

### Electrochemical Measurement

Electrochemical analysis was fulfilled by the DyneChem electrochemical workstation, and platinum and Ag/AgCl are used as the counter electrode and reference electrode, respectively. The cyclic voltammogram and tafel polarization curves were tested in 2 M polysulfide electrolyte. Nyquist plots of QDSSCs are under the illumination of one full sun intensity. The voltage used during the EIS measurements is 0.5 V.

### Characterization

The surface morphology was analyzed by scanning electron microscopy (SEM) (JEOL7610), the characterization of samples was measured by X-ray photoelectron spectroscopy (XPS) (Kratos Axis UltraDLD), and the *J*–*V* curves of QDSSCs were measured by Keithley 2400 source meter (Zolix Instruments Co., Ltd.). IR temperature images are obtained by FLIR T460.

## Results and Discussion

**Fig. 2:**
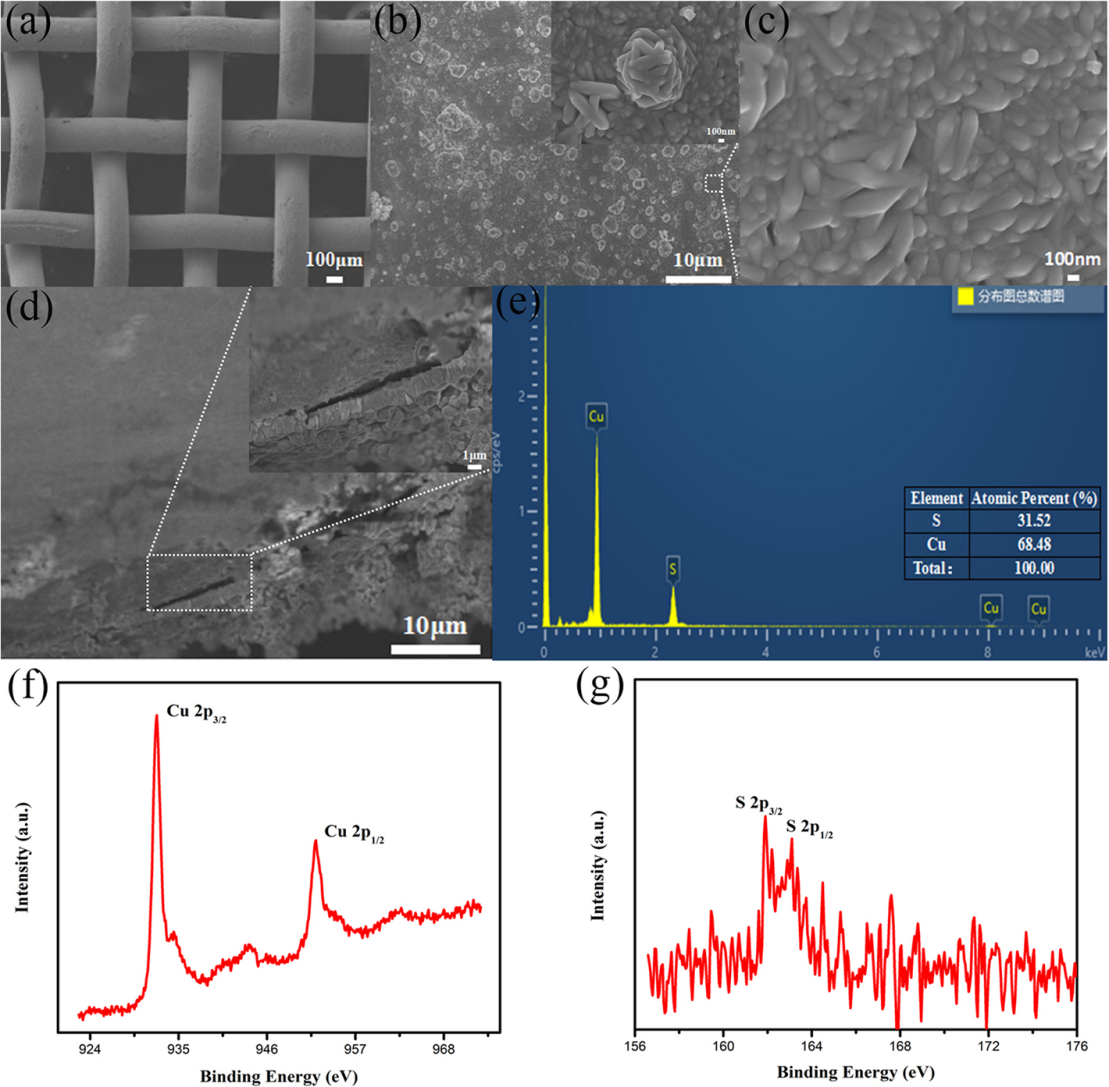
SEM images of the mesh-structured Cu_2_S counter electrode: **a** top view; **b**, **c** enlarged view of surface morphology; **d** cross-sectional view and enlarged view of the Cu_2_S layer. **e** EDS of Cu_2_S. **f**, **g** XPS survey spectrums of Cu_2_S samples

There are numerous nanostructured materials that can be used as counter electrodes for QDSSCs, including Cu_2_S, CoS, GeC [35], and NCW [36]. However, the Cu_2_S counter electrode based on brass mesh exhibits ideal catalytic activity toward Sn^2−^/S^2−^ redox couple. To prepare the Cu_2_S counter electrode, two different methods are used. One is to remove Zn from the surface of brass mesh with hydrochloric acid followed by immersing the mesh in a mixed solution containing CH_4_N_2_S and C_2_H_8_N_4_ for 24 h [37]. The SEM images of resulted Cu_2_S covered mesh are shown in Fig. 2a–c. The magnified SEM image indicates the granular-structured Cu_2_S are formed. The cross-sectional view in Fig. 2d indicates the existence of Cu_2_S film on the brass wire, the crack between them originates from cutting during the preparation of the cross-sectional sample, and the thickness is about 1.3 μ3oss-sectional samp in Fig. 2e reveals that the only elements that exist there are Cu and S, and the atom ration of Cu to S is approximately 2. The XPS spectra of Cu and S are shown in Fig. 2f and h, respectively. The peaks of Cu 2p at 952 eV and 932.5ev correspond to the electron binding energy of Cu 2p1/2 and Cu 2p3/2, respectively, which is consistent with the Cu^1+^ [38]. The peaks observed in S 2p at 163.5 eV and 161 eV correspond to S 2p1/2 and S 2p3/2, respectively, which is consistent with S^2−^ [39]. These results confirm the successful preparation of Cu_2_S film on the brass mesh, and the surface morphology is supposed to ensure its large surface area and sufficient catalytic activity.

The other method has been extensively used in previous reports, in which the brass mesh is firstly placed in hydrochloric acid, then it is subsequently dipped into the polysulfide electrolyte (1 M Na_2_S and 1 M S) for a suitable time [40]. The corresponding results are shown in Fig. S2. The magnified SEM image indicates the flake-structured Cu_2_S are formed. The cross-sectional view indicates the existence of Cu_2_S film on the brass wire, and the thickness is also about 1.3 μ.3sThe Cu XPS spectra show peaks at 933.1 eV and 952.5 eV, corresponding to Cu 2p3/2 and 2p1/2, respectively [41]. The S 2p spectrum shows a peak at 162.4 eV, which confirms the presence of S^2−^ [42]. The effect of Cu_2_S morphology on cell performance is further investigated.

**Fig. 3:**
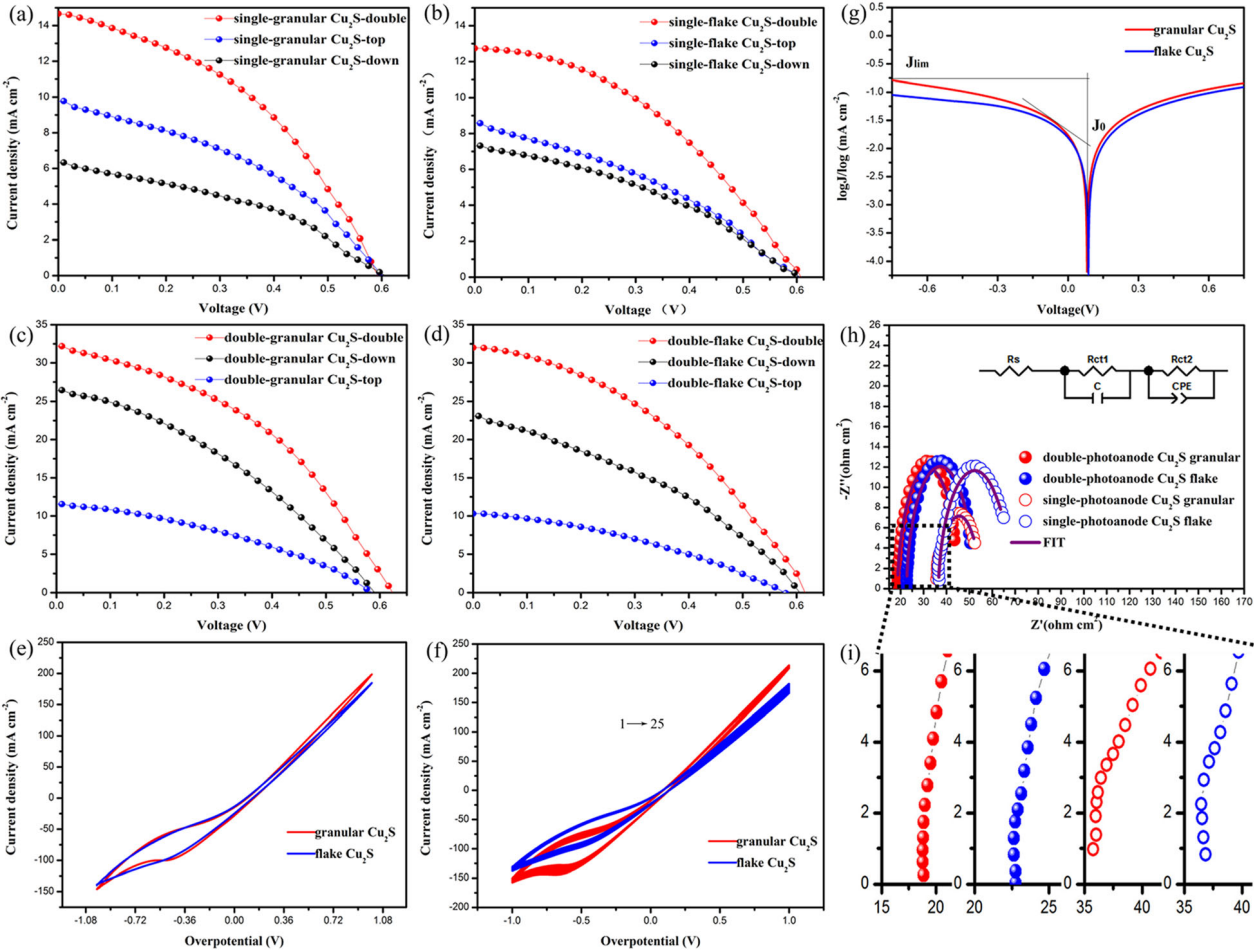
Champion *J*–*V* curves of QDSSCs with different structures. **a** Single-photoanode and granular Cu_2_S CE. **b** Single-photoanode and flake Cu_2_S CE. **c** Double-photoanode and granular Cu_2_S CE. **d** Double-photoanode and flake Cu_2_S CE. **e** CV and **g** tafel polarization curves of two Cu_2_S CEs, respectively. **f** Electrochemical stability of various CEs studied by CV. **h** Nyquist plots of QDSSCs with different CEs and different structures under the illumination of one full sun intensity. **i** Enlarged part of the black dotted frame in **h**

**Fig. 4:**
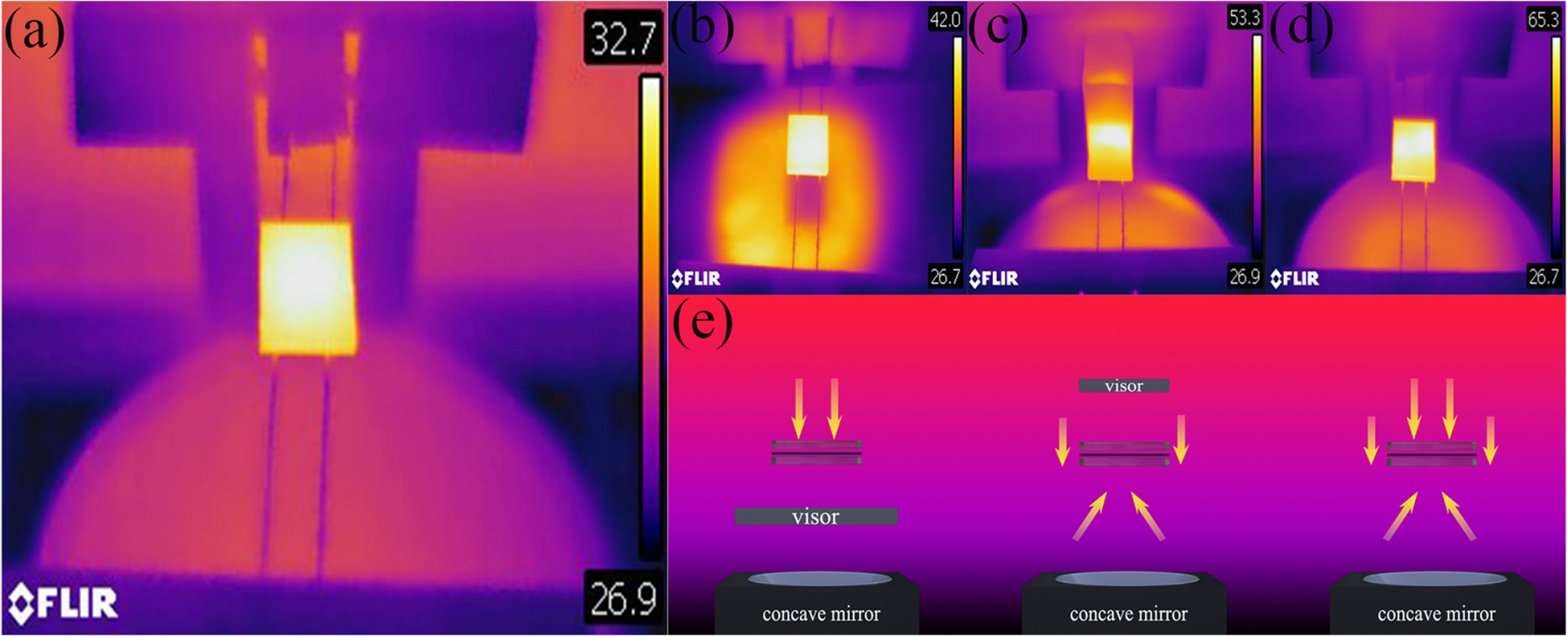
Infrared photographs of different lighting conditions: **a** without light, **b** parallel light on the upper side, **c** concentrated light from the parabolic reflector, **d** light from both sides shines simultaneously, and **e** simulation diagram of different lighting conditions

To characterize the performance of cells with different architectures and CEs, the photocurrent density–voltage (*J*–*V*) curves are acquired and shown in Fig. 3a–d. Single photoanode device and double photoanode device are both fabricated for comparison with granular and flake Cu_2_S as CEs, respectively. There are three ways for illuminating the cells: top, bottom, and double directions as shown in Fig. 4e, and the behaviors of cell working under different illumination conditions are investigated in detail, and the corresponding cell parameters are summarized in Table 1 including open-circuit voltage (*V*_oc_), short-circuit current (*J*_sc_), filling factor (FF), and PCE, in which the values not in brackets are the experimental average of twenty group cells and those in brackets are champion values. The test configuration for the single photoanode device with granular and flake Cu_2_S as CEs is shown in Fig. S3, and the *J*–*V* curves are shown in Fig. 3a and b. The PCE irradiating from the top, down, and double directions are 2.32%, 2.00%, and 3.59%, respectively, while the *V*_oc_ are kept around 0.6 V, and the main increase in PCE is due to elevated *J*_sc_. Since this is a single photoanode device, the increased *J*_sc_ should be due to the augmented light intensity from bottom irradiation, which results in increased photo-generated electrons in photoanodes. It should be noticed that the PCE of down irradiation is lower than that of top irradiation even if the concentrated light is higher in intensity than parallel light from the top, which could get an explanation from Fig. S3. When concentrating light on single photoanode QDSSCs, the light needs to pass through FTO glass, then through the Cu_2_S mesh CE and electrolyte to reach the top photoanode, inducing photoenergy loss to some extent. From our result, the intensity of photoenergy arriving the top photoanode would be weaker than parallel light irradiation from the top. Therefore, the PCE of the single photoanode cell structure under concentrated light irradiation is lower than that under parallel light irradiation on the upper side. It could be further supported by adopting test configuration shown in Fig. S4. Focusing light directly onto the bottom photoanode as working electrode achieves a PCE of 4.32% and a short-circuit current of 21.6 mA cm^−2^, almost twice the short-circuit current generated by the parallel light irradiating the photoanode, which means that the photon flux after condensing is twice that of the parallel light from the top. From the change of photon flux, we can calculate the condensing coefficient is 2, which is consistent with our measured results. As can be seen from Table 1, the average PCE of the double photoanode cell structure with granular Cu_2_S CE is 5.51% under the condition of concentrating light from the bottom side, and the increase in PCE is due to the contribution of top photoelectrode, indicating that the top photoanode captures more photons under the concentrating condition.

**Table 1:** Parameters obtained from *J*–*V* curves of various QDSSCs under different lighting conditions. The values not in brackets are the experimental average of twenty group cells, and those in brackets are champion values

Cell	*V*_oc_ [V]	*J*_sc_ [mA cm^−2^]	FF [%]	PCE [%]
Single-granular Cu_2_S-top	0.583 (0.602)	9.682 (9.760)	0.394 (0.430)	2.18 (2.32)
Single-granular Cu_2_S-down	0.599 (0.606)	6.256 (6.336)	0.407 (0.409)	1.91 (2.00)
Single-granular Cu_2_S-double	0.584 (0.592)	14.663 (14.681)	0.402 (0.413)	3.58 (3.59)
Single-flake Cu_2_S-top	0.604 (0.610)	8.969 (8.978)	0.381 (0.401)	1.94 (1.98)
Single-flake Cu_2_S-down	0.594 (0.607)	7.314 (7.350)	0.401 (0.410)	1.78 (1.79)
Single-flake Cu_2_S-double	0.590 (0.610)	13.294 (13.308)	0.378 (0.397)	2.92 (3.09)
Double-granular Cu_2_S-top	0.573 (0.585)	11.550 (11.552)	0.391 (0.399)	2.55 (2.64)
Double-granular Cu_2_S-down	0.582 (0.590)	26.287 (26.332)	0.353 (0.356)	5.51 (5.53)
Double-granular Cu_2_S-double	0.616 (0.629)	32.247 (32.297)	0.395 (0.408)	8.17 (8.28)
Double-flake Cu_2_S-top	0.561 (0.578)	10.311 (10.418)	0.356 (0.347)	2.12 (2.21)
Double-flake Cu_2_S-down	0.586 (0.605)	22.956 (23.045)	0.336 (0.359)	4.87 (5.00)
Double-flake Cu_2_S-double	0.616 (0.624)	32.001 (32.012)	0.396 (0.404)	7.80 (7.68)

For the double photoanode device with granular Cu_2_S as CEs, the PCE irradiating from the top, down, and double directions are 2.64% (*J*_sc_ = 11.552 mA cm^−2^), 5.53% (*J*_sc_ = 26.287 mA cm^−2^), and 8.28% (*J*_sc_ = 32.247 mA cm^−2^), respectively. Compared with that of traditional single photoanode structure irradiated from the top (2.32%), the PCE increases by 260%. The increase in PCE is obviously due to the increased *J*_sc_, indicating the double photoanodes work in a parallel configuration. The measured light intensity of the concentrated light from the bottom is approximately twice that of the upper parallel light. Under the irradiation condition of the concentrated light, the PCE of QDSSCs should be higher than that of the upper parallel light. It is consistent with the present result that the PCE from bottom irradiation is more than 2 times compared with that from top irradiation.

It should be noticed that the behaviors of cells based on flake Cu_2_S CE are similar but showing overall decayed values in photovoltaic parameters. To explore the detailed origination, the cyclic voltammogram (CV) curves and tafel polarization curves of the two kids of Cu_2_S CEs are measured using a three-electrode system. Figure 3e is the CV curves, and granular Cu_2_S shows higher current density. The reduction peak of granular Cu_2_S is higher than flake Cu_2_S, indicating the faster redox reaction rate of S^2−^/S_n_^2−^ in the electrolyte. Figure 3f shows the CV curves of the Cu_2_S electrode with different morphologies from 1 to 25 cycles. There is no obvious variation that could be found for the shape of the CV curve and the position of the peak. The CV curves overlap well under continuous scanning, indicating that the two morphologies of the electrode both own good chemical stability. It can be seen from Fig. 3g that the exchange current density (*J*_0_) of granular Cu_2_S is larger than that of flake Cu_2_S. According to the test results of CV and tafel polarization curves, it suggests that the catalytic activity of granular Cu_2_S is better than that of Cu_2_S film. This is consistent with the photovoltaic performance of *J*–*V* curves in Fig. 3a–d. To further confirm the catalytic activity of the counter electrodes, EIS spectra are studied and shown in Fig. 3h. The purple solid lines represent fitting curves, and the fitting data are summarized in Table 2. *R*_ct1_, *R*_ct2_, and *R*_s_ represent charge transfer resistance on the counter electrode, charge transfer resistance at the photoanode/electrolyte interface, and series resistance, respectively. It can be seen that the structure of a single photoanode cell and the charge transfer resistances of *R*_ct1_ for granular Cu_2_S and flake Cu_2_S is 3.522 and 6.753 Ω cm^2^, respectively, and those of double photoanode cell are 5.990 and 8.088 Ω cm^2^, respectively. The smaller *R*_ct1_ of granular Cu_2_S indicates better conductivity due to its smaller charge transfer resistance.

**Table 2:** Fitted values of *R*_S_, *R*_ct1_, and *R*_ct2_ of QDSSCs with different structures and morphologies from the Nyquist plots

Counter electrode	*R*_s_ (Ω)	*R*_ct1_ (Ω cm^2^)	*R*_ct2_ (Ω cm^2^)
Double-photoanode Cu_2_S granular	18.65	5.990	20.81
Double-photoanode Cu_2_S flake	22.63	8.088	21.42
Single-photoanode Cu_2_S granular	35.62	3.522	15.36
Single-photoanode Cu_2_S flake	36.47	6.753	25.03

The photon flux inside an absorber conforms to Lambert–Beer law:

1 $$ b\left(E,x\right)=\left[1-R(E)\right] bs(E)\exp \left[-{\int}_0^x\alpha \left(E,x\hbox{'}\right) dx\hbox{'}\right] $$

where *b*(*E*, *x*) is the photon flux received by QDs at position *x*, *bs*(*E*) is the photon flux received in the vertical direction to solar irradiation, *R*(*E*) is the reflectivity of the interface, and *α*(*E*, *x*) is the absorption coefficient at position *x*. CPV collect incident light over a large area and focus it on a small area of solar cell for photovoltaic conversion. Concentration factor *X* is an important parameter to describe the performance of concentrating solar cells, which is the increased times of photon flux density *b*(*E*) and is also approximated to be the ratio of the area of collected incident light to the area of solar cells. It could be considered that the focused light is uniform within the half angle of concentration (*θ*_*X*_), and the increased times of solar photon flux *b*_s_(*E*,*x*) equals to concentration factor *X*. Therefore, for CPV, Eq. (1) could be expressed as:

2 $$ b\left(E,x\right)=\left[1-R(E)\right]\cdot X\cdot bs(E)\exp \left[-{\int}_0^x\alpha \left(E,x\hbox{'}\right) dx\hbox{'}\right] $$

The photocurrent (*J*_ph_) generated by a solar cell under light conditions, determined by the photon flux and the performance of the solar cell, *J*_ph_ is equal to *J*_sc_, and can be expressed as:

3 $$ J\mathrm{ph}=J\mathrm{sc}=q{\int}_0^{\infty}\mathrm{QE}(E) bs\left(E, Ts\right) dE $$

where *Q* is the elementary charge. quantum efficiency (QE) is a function of photon energy. We assume that the photon flux of the collector *bs*_2_ is *Y* times that of the top photon flux *bs*_1_, and the *J*_sc_ of the double photoanode cell under irradiation from the down side could be expressed as:

4 $$ J\mathrm{sc}(Ybs)=q{\int}_0^{\infty}\mathrm{Q}E(E)\cdot Y\cdot bs\left(E, Ts\right) dE\approx {YJ}_{\mathrm{sc}} $$

Therefore, the sum of *J*_sc1_ value under the condition of parallel light irradiation from the top side and *J*_sc2_ value under the condition of concentrated light irradiation from the down side should be equal to *J*_sc1, 2_ generated by light irradiation on both sides of the PV. Equation (4) is basically consistent with *J*–*V* results in Table 1, however with some deviations. As can be seen from the *J*_sc_ data, the sum of *J*_sc1_ illuminating from the top and *J*_sc2_ illuminating from the down is a little smaller than that of *J*_sc1, 2_ illuminating from both sides for double photoanode devices. The deviation should result from the photothermal effect due to the temperature variation of QDSSCs under different illuminating conditions.

It is well known that the focused light on optoelectrical devices would induce thermal accumulation and increased temperature. In the present work, the photon flux of the concentrated light from the parabolic reflector measured by the light intensity meter is approximately twice the normal sunlight simulator; it means that the concentration coefficient is 2, which is much smaller than practical CPV devices. However, it is still necessary to investigate the effect of thermal accumulation on the cell. We use an infrared (IR) camera to monitor the temperature variation under different testing conditions. The focal length of the used parabolic reflector is 6.5 cm. We place the photovoltaic device 0.5 cm below the focus point of the parabolic reflector to ensure the full illumination of the active area, which is about 1.5 cm^2^. The IR temperature images for double photoanode devices are shown in Fig. 4b–d, and the corresponding irradiation conditions are shown in Fig. 4e. Compared with non-irradiated devices, the temperature increases are 9.3 °C, 20.6 °C, and 32.6 °C, respectively. The double side irradiation shows a higher temperature increase than the sum of separate irradiation from the top and down, which indicates the additional mechanism from the material or interface involved here. The reaction of S^2−^/S_n_^2−^ in electrolyte and interface is not similar under different temperatures. According to the Arrhenius formula, the chemical reaction rate in PV increases with the increase of temperature. With the increase of temperature, the redox reaction near the QDSSC electrodes was enhanced, which accelerated the depletion of photogenic holes in the photoanode, thus greatly reducing the energy-excited redox reaction.

## Conclusions

In summary, we design a new solar cell architecture by integrating the CPV concept into QDSSCs with double photoanode design. To ensure the better light harvesting, the Cu_2_S mesh is used as a counter electrode and sandwiched between two photoanodes, and the effect of Cu_2_S morphology on cell performance is investigated and optimized. It has been shown that the special design achieved a 260% enhancement in PCE compared with the single photoanode irradiated from the top side. While the open voltage circuit does not change, the double photoanode design greatly increases the current density and thus increases PCE. In addition, the photothermal effect induced by CPV could be helpful to improve PCE, and a champion PCE of 8.28% (*V*_oc_ = 0.629 V, *J*_sc_ = 32.247 mA cm^−2^) is achieved. It should be mentioned that the present does provide a new way for boosting PCE of QDSSCs, but the device design specially for the CPV part could be further optimized, and the performance of the cell would be further enhanced. We believe that with better QD sensitizers and device design techniques, the idea suggested in the present work would induce great progress in QDSSCs and could be expanded to other solar cells.

## Supplementary information

**Additional file 1: Figure S1.**The cross-section SEM of TiO_2_ photoanode. **Figure S2.** SEM top view (a) and cross-sectional view (b) of flake Cu_2_S; (c) and (d) are XPS spectra of Cu and S. The XPS spectra of as-prepared flake Cu_2_S CEs show peaks at 933.1 eV and 952.5 eV, corresponding to Cu 2p3/2 and 2p1/2, respectively. The S 2p spectrum shows a peak at 162.4 eV, which confirms the presence of S^2-^. **Figure S3**. Schematic illustration of a single-photoanode structure for J-V measurement irradiated from downside. It needs to penetrate the bottom photoanode, electrolyte and Cu_2_S mesh structure to arrive the top photoanode. **Figure S4.** When the light is condensed by a parabolic reflector and irradiates from the bottom photoanode, it can generate a short circuit current of about 21.6 mA cm^-2^, almost twice the short-circuit current generated by the parallel light irradiating the photoanode, which means that the photon flux after condensing is twice that of the parallel light from top. From the change of photon flux, we can calculate the condensing coefficient is 2, which is consistent with our measured results. **Figure S5.** The PCE of cells using the Cu_2_S mesh CE composed of 0.22 mm copper wires was 6.79% and 6.25%, respectively. The diameter of the Cu wire is inversely related to the aperture of the Cu mesh. In this work, the diameter of the Cu mesh used is 0.25 mm. The PCE results for a Cu mesh CE with wire diameter of 0.22 mm are shown in Figure S5, which is are lower than that of 0.25 mm Cu mesh.

## Data Availability

The datasets used or analyzed during the current study are available from the corresponding author on reasonable request.

## Abbreviations

QDSSCs: Quantum dot-sensitized solar cells
PCE: Power conversion efficiency
CPV: Concentrating solar cell
QDs: Quantum dots
FF: Fill factor
SILAR: Successive ionic layer adsorption and reaction
CV: Cyclic voltammetry
CBD: Chemical bath deposition
SEM: Scanning electron microscopy
XPS: X-ray photoelectron spectra
