# Supplementary material for: Boosting Power Conversion Efficiency of Quantum Dot-Sensitized Solar Cells by Integrating Concentrating Photovoltaic Concept with Double Photoanodes
Source: Nanoscale Res Lett. 2020 Sep 29;15:188. doi: 10.1186/s11671-020-03424-8 (PMC7524932; doi:10.1186/s11671-020-03424-8)
Supplement: Supplementary file 1 — Additional file 1: Figure S1.The cross-section SEM of TiO2 photoanode. Figure S2. SEM top view (a) and cross-sectional view (b) of flake Cu2S; (c) and (d) are XPS spectra of Cu and S. The XPS spectra of as-prepared flake Cu2S CEs show peaks at 933.1 eV and 952.5 eV, corresponding to Cu 2p3/2 and 2p1/2, respectively. The S 2p spectrum shows a peak at 162.4 eV, which confirms the presence of S2-. Figure S3. Schematic illustration of a single-photoanode structure for J-V measurement irradiated from downside. It needs to penetrate the bottom photoanode, electrolyte and Cu2S mesh structure to arrive the top photoanode. Figure S4. When the light is condensed by a parabolic reflector and irradiates from the bottom photoanode, it can generate a short circuit current of about 21.6 mA cm-2, almost twice the short-circuit current generated by the parallel light irradiating the photoanode, which means that the photon flux after condensing is twice that of the parallel light from top. From the change of photon flux, we can calculate the condensing coefficient is 2, which is consistent with our measured results. Figure S5. The PCE of cells using the Cu2S mesh CE composed of 0.22 mm copper wires was 6.79% and 6.25%, respectively. The diameter of the Cu wire is inversely related to the aperture of the Cu mesh. In this work, the diameter of the Cu mesh used is 0.25 mm. The PCE results for a Cu mesh CE with wire diameter of 0.22 mm are shown in Figure S5, which is are lower than that of 0.25 mm Cu mesh. [file 11671_2020_3424_MOESM1_ESM.docx]

Supplementary Material

**Boosting power conversion efficiency of quantum dot sensitized solar cells by integrating concentrating photovoltaic concept with double photoanodes**

Pei Xu^a^, Xiaopeng Chang^a^, Runru Liu^a,^*, Liying Wang^b^, Xuesong Li^b^, Xueyu Zhang^b^, Xijia Yang^b^, Dejun Wang^a^, and Wei Lü^b,^*

^a^ Key Laboratory of Materials Design and Quantum Simulation, College of Science

Changchun University, Changchun 130012, P. R. China

^b^ Key Laboratory of Advanced Structural Materials, Ministry of Education & Advanced Institute of Materials Science ,Changchun University of Technology, Changchun 130012, P. R. China


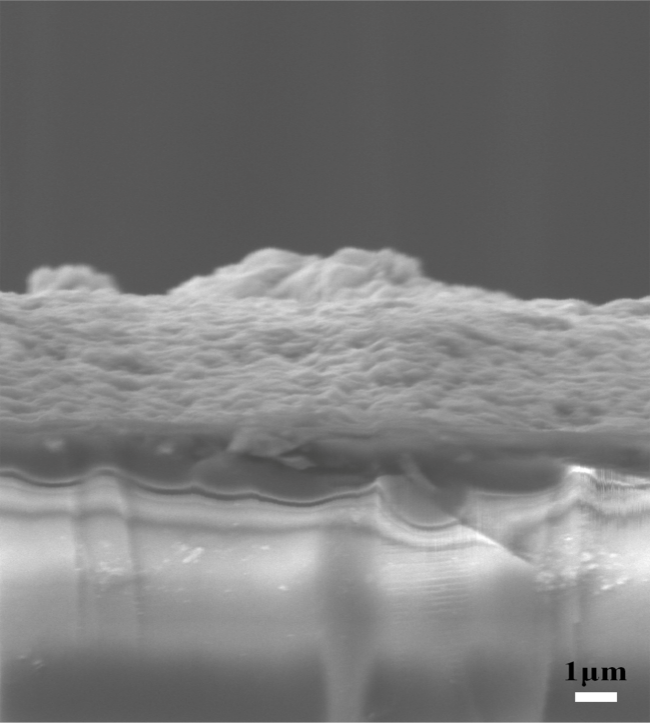


**Figure S1.**The cross-section SEM of TiO_2_ photoanode.


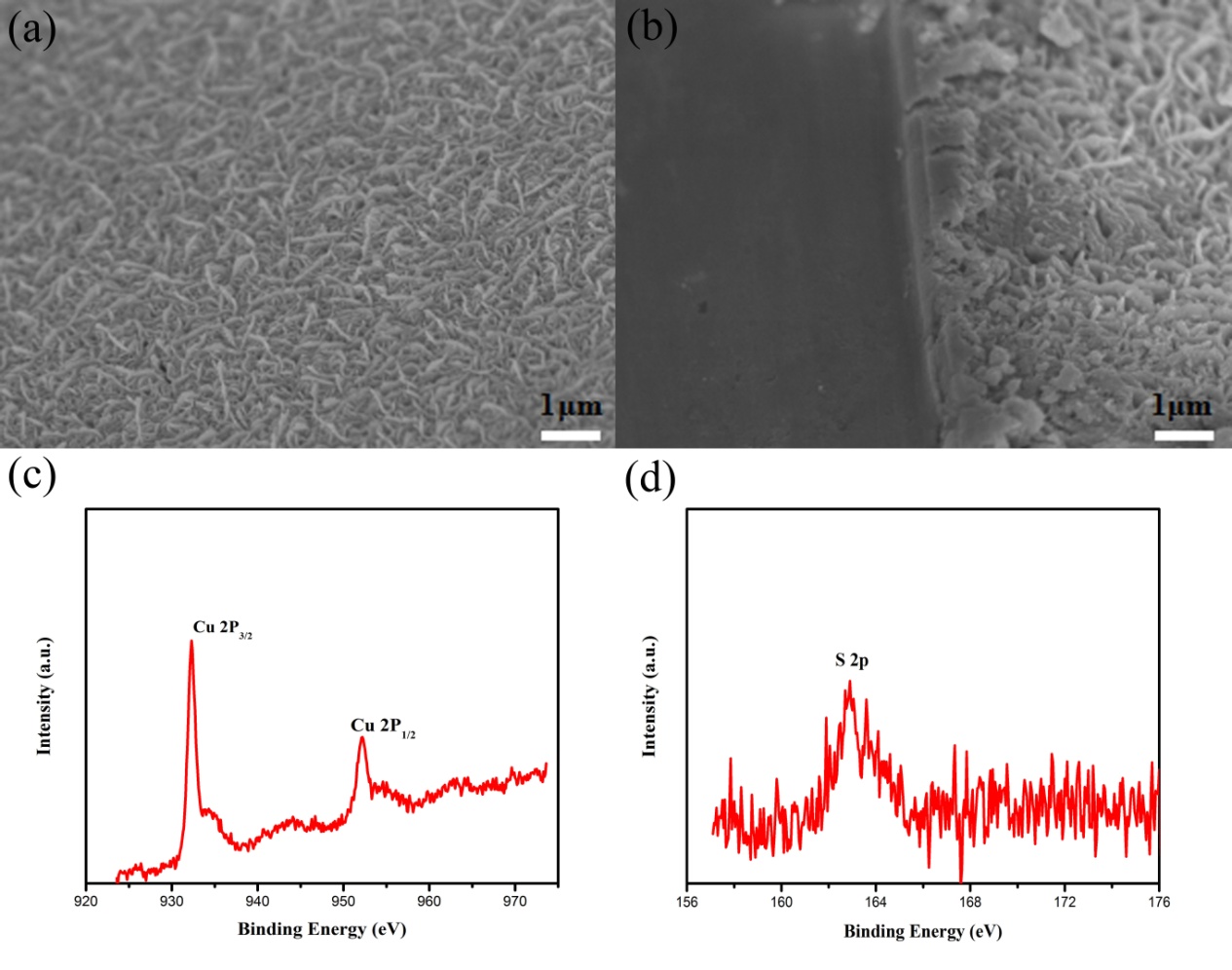


**Figure S2.** SEM top view (a) and cross-sectional view (b) of flake Cu_2_S; (c) and (d) are XPS spectra of Cu and S. The XPS spectra of as-prepared flake Cu_2_S CEs show peaks at 933.1 eV and 952.5 eV, corresponding to Cu 2p3/2 and 2p1/2, respectively. The S 2p spectrum shows a peak at 162.4 eV, which confirms the presence of S^2-^.


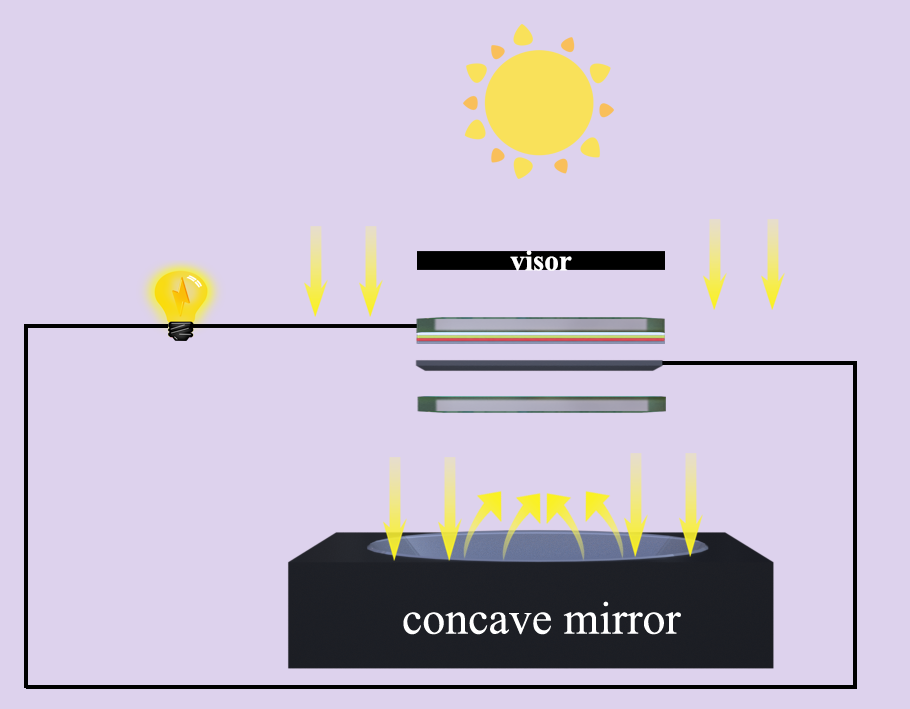


**Figure S3**. Schematic illustration of a single-photoanode structure for J-V measurement irradiated from downside. It needs to penetrate the bottom photoanode, electrolyte and Cu_2_S mesh structure to arrive the top photoanode.


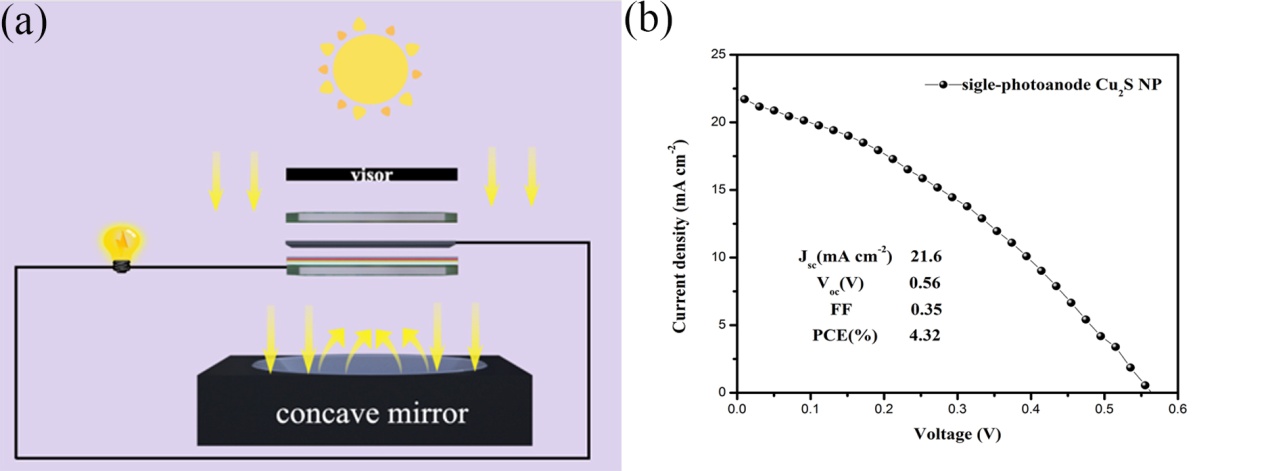


**Figure S4.** When the light is condensed by a parabolic reflector and irradiates from the bottom photoanode, it can generate a short circuit current of about 21.6 mA cm^-2^, almost twice the short-circuit current generated by the parallel light irradiating the photoanode, which means that the photon flux after condensing is twice that of the parallel light from top. From the change of photon flux, we can calculate the condensing coefficient is 2, which is consistent with our measured results.


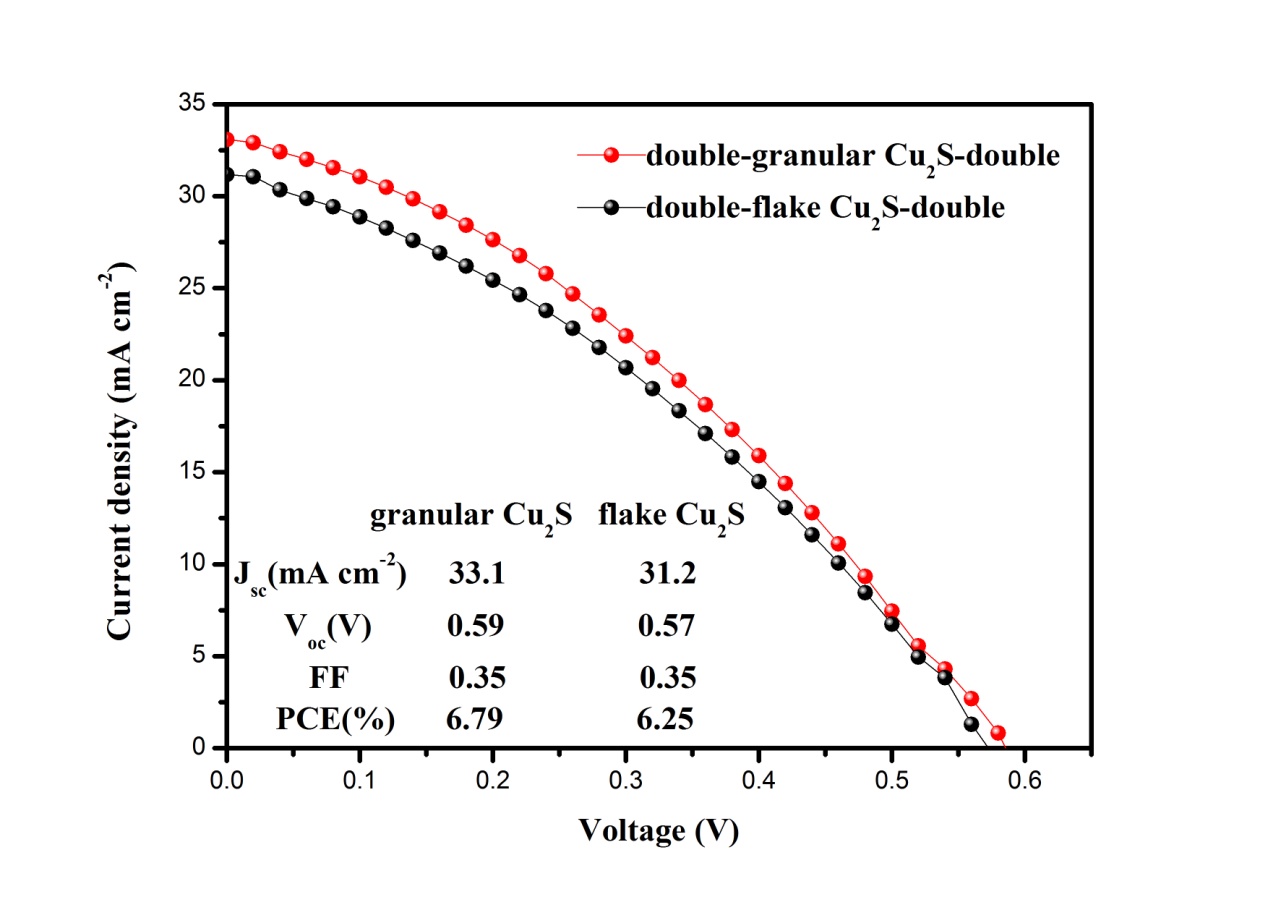


**Figure S5.** The PCE of cells using the Cu_2_S mesh CE composed of 0.22 mm copper wires was 6.79% and 6.25%, respectively. The diameter of the Cu wire is inversely related to the aperture of the Cu mesh. In this work, the diameter of the Cu mesh used is 0.25 mm. The PCE results for a Cu mesh CE with wire diameter of 0.22 mm are shown in Figure S5, which is are lower than that of 0.25 mm Cu mesh.
